# One-year functional outcome of the Flying Intervention Team versus patient interhospital transfer in acute ischaemic stroke

**DOI:** 10.1093/esj/aakag025

**Published:** 2026-04-06

**Authors:** Nikolai D Hubert, Markus Holler, Saskia R Wernsdorf, Sophie Herdegen, Christian Maegerlein, Hanni Wiestler, Lucie Esterl-Pfäffl, Dennis Dietrich, Thomas Witton-Davies, Isabel Heinrich, Anastasios Mpotsaris, Philip M Bath, Heinrich J Audebert, Roman L Haberl, Gordian J Hubert

**Affiliations:** TEMPiS Telestroke Center, Department of Neurology, München Klinik, Academic Teaching Hospital of the Ludwig-Maximilians-University, Sanatoriumsplatz 2, Munich 81545, Germany; TEMPiS Telestroke Center, Department of Neurology, München Klinik, Academic Teaching Hospital of the Ludwig-Maximilians-University, Sanatoriumsplatz 2, Munich 81545, Germany; TEMPiS Telestroke Center, Department of Neurology, München Klinik, Academic Teaching Hospital of the Ludwig-Maximilians-University, Sanatoriumsplatz 2, Munich 81545, Germany; TEMPiS Telestroke Center, Department of Neurology, München Klinik, Academic Teaching Hospital of the Ludwig-Maximilians-University, Sanatoriumsplatz 2, Munich 81545, Germany; TUM School of Medicine and Health, Klinikum Rechts der Isar, Technical University of Munich (TUM), Ismaninger Str. 22, Munich 81675, Germany; TEMPiS Telestroke Center, Department of Neurology, München Klinik, Academic Teaching Hospital of the Ludwig-Maximilians-University, Sanatoriumsplatz 2, Munich 81545, Germany; TEMPiS Telestroke Center, Department of Neurology, München Klinik, Academic Teaching Hospital of the Ludwig-Maximilians-University, Sanatoriumsplatz 2, Munich 81545, Germany; TEMPiS Telestroke Center, Department of Neurology, München Klinik, Academic Teaching Hospital of the Ludwig-Maximilians-University, Sanatoriumsplatz 2, Munich 81545, Germany; Department of Diagnostic and Interventional Radiology, München Klinik, Sanatoriumsplatz 2, Munich 81545, Germany; TEMPiS Telestroke Center, Department of Neurology, München Klinik, Academic Teaching Hospital of the Ludwig-Maximilians-University, Sanatoriumsplatz 2, Munich 81545, Germany; Department of Diagnostic and Interventional Radiology, München Klinik, Sanatoriumsplatz 2, Munich 81545, Germany; Stroke Trials Unit, Mental Health & Clinical Neuroscience, University of Nottingham, Nottingham NG7 2UH, United Kingdom; Department of Neurology, Charité Universitätsmedizin Berlin, corporate member of Freie Universität Berlin, Humboldt-Universität zu Berlin and Berlin Institute of Health, Campus Benjamin Franklin, Hindenburgdamm 30, Berlin 12200, Germany; Center for Stroke Research Berlin, corporate member of Freie Universität Berlin, Humboldt-Universität zu Berlin and Berlin Institute of Health, Charité Universitätsmedizin Berlin, Charitéplatz 1, Berlin 10117, Germany; TEMPiS Telestroke Center, Department of Neurology, München Klinik, Academic Teaching Hospital of the Ludwig-Maximilians-University, Sanatoriumsplatz 2, Munich 81545, Germany; TEMPiS Telestroke Center, Department of Neurology, München Klinik, Academic Teaching Hospital of the Ludwig-Maximilians-University, Sanatoriumsplatz 2, Munich 81545, Germany

**Keywords:** acute ischaemic stroke, mechanical thrombectomy, mobile intervention team, stroke systems of care, telestroke, time is brain

## Abstract

**Introduction:**

Endovascular thrombectomy is highly effective for large vessel occlusion strokes, yet timely access remains challenging in remote areas. The Flying Intervention Team study demonstrated that dispatching a neurointerventionist team to peripheral hospitals reduces time to treatment. This study evaluates 12-month functional outcomes of enrolled patients.

**Patients and methods:**

This is a secondary analysis of a multicentre pseudo-randomised controlled intervention study comparing 2 systems of care in alternating weeks. The study was conducted in 13 nonurban primary stroke centres in Bavaria, Germany. Of 157 patients enrolled between February 2018 and October 2019, 146 had available 12-month follow-up information. Patients were treated either by the flying team or after interhospital transfer to a referral centre. Primary outcome was the modified Rankin Scale (mRS) score at 12 months in the intention-to-treat analysis. Secondary outcomes included quality of life, activities of daily living and mortality.

**Results:**

Overall, 146 patients were included (median [IQR] age, 75 [66–80] years; 79 [54%] women), 70 in the flying team group and 76 in the transfer group. Median decision-to-puncture time was 89 min shorter in the flying team group. No significant differences were found in successful reperfusion and complication rates. mRS scores at 12 months favoured the flying team (3 [IQR 1–6] vs 4 [2–6]; adjusted common odds ratio, 2.03; 95% CI, 1.09–3.84). Surviving patients had significantly better quality of life (EQ-5D mean [SD] utility score, 0.79 [0.24] vs 0.68 [0.31]). No significant differences were observed in Barthel index or mortality.

**Discussion and conclusion:**

In rural stroke patients, deployment of a flying team was associated with better functional outcomes at 12 months. These findings reinforce the potential for broader implementation of the model to increase equitable access to stroke care.

## Graphical Abstract

**Graphical Abstract:**
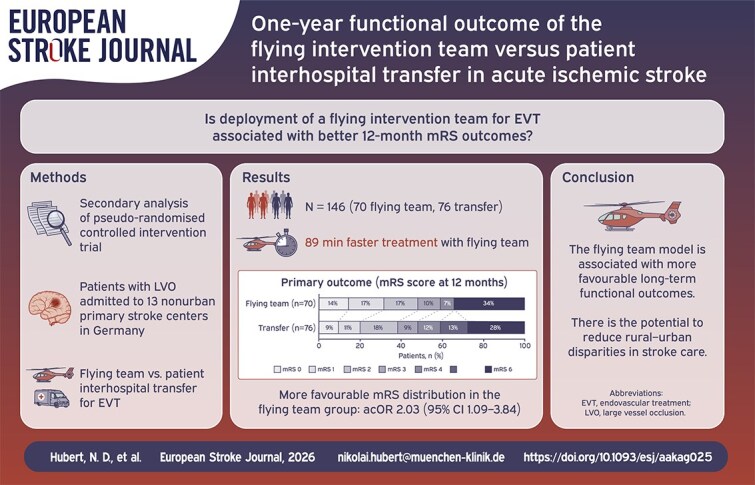

## Introduction

Endovascular thrombectomy (EVT) is a highly effective treatment for patients with acute ischaemic stroke caused by large vessel occlusion (LVO),^1^ particularly when performed early.^2^ However, urban–rural disparities exist regarding time delay until treatment.^3–6^ Door-in-door-out times at primary stroke centres and long-distance transportation delays to comprehensive stroke centres are the main reasons for these disparities.^7^ The Flying Intervention Team (FIT) project was developed to reduce the difference in treatment time.^8^ In this model of care, suspected stroke patients are initially admitted to the closest local stroke centre for rapid intravenous thrombolysis treatment and identification of LVO. After telemedical evaluation indicating suitability for EVT, a neurointervention team from a comprehensive stroke centre is dispatched via helicopter to the local stroke centre to perform EVT on site. While the flying team is on the way, the patient is prepared in the local angiography suite for acute intervention.^8^ A controlled intervention study demonstrated that this system of care reduced time to EVT by 90 min compared with secondary patient transfer to a comprehensive centre during weeks when no FIT service was available.^9^ A more favourable functional outcome at 3 months was observed in patients receiving EVT in the flying team group, with a statistically significant difference in the intention-to-treat analysis including all patients initially deemed suitable for EVT. However, short-term follow-up may not fully capture the benefits of earlier reperfusion. Assessing outcomes at 12 months may therefore be valuable for clinical decision-making and healthcare policy.

The aim of this study was to investigate the association of the Flying Intervention Team and functional outcome at 12 months after stroke.

## Patients and methods

### Study design

This secondary analysis was based on a pseudo-randomised controlled intervention study comparing two systems of care in alternating weeks between 1 February 2018 and 24 October 2019. The study was registered at ClinicalTrials.gov, NCT04270513. The study design is described elsewhere.^9^ In brief, acute ischaemic stroke patients admitted to primary stroke centres and designated for EVT were either treated by the flying team at the primary stroke centre or at a referral centre following patient interhospital transfer. The flying team service was available 26 weeks per year, from 8 AM to 10 PM, including weekends. During all other weeks, patient interhospital transfer was organised, and patients were included in the study during the same service times from 8 AM to 10 PM. The allocation of the flying team and transfer weeks was preplanned, alternating and maintained at a 1:1 ratio. The allocation schedule was predefined and structured to ensure a balanced distribution of intervention and transfer weeks across the entire study period, thereby minimising potential seasonal imbalance. Owing to a large difference in the primary outcome (time delay until treatment) in favour of the flying team, the ethics committee stopped the study after a preplanned interim analysis.

### Study population

A total of 13 regional hospitals of the Telemedical Stroke Network in Southeast Bavaria (TEMPiS) participated in the study (Figure S1). The TEMPiS network provides teleconsultations, quality management and training programmes. All hospitals had a local stroke unit, intensive care unit, helicopter pad and angiography suite or cardiac catheter lab. Both anaesthesia support and an angiography assistant were available on site. Consecutive patients for whom a decision was made to pursue EVT were included, and the predefined allocated system of care was deployed. The inclusion criteria were age 18–85 years, occlusion of the M1 or proximal M2 segment of the middle cerebral artery, intracranial internal carotid artery (ICA) or basilar artery, decision to carry out intervention between 8 AM and 10 PM, presentation within the predefined time window (<6 h in anterior circulation, < 24 h in basilar artery occlusion, < 24 h in anterior circulation with a relevant mismatch detected via perfusion imaging), no severely decreased life expectancy, an Alberta Stroke Program Early CT (ASPECT) Score ≥ 6 and a premorbid mRS score ≤ 3. Patients without available 12-month follow-up data were excluded from the secondary analysis.

### Exposures

The team on call consisted of a neurointerventionist and an angiography assistant, both of whom were trained on all the angiography suites of the participating hospitals. Training included device-specific instruction in accordance with national regulatory requirements and manufacturer standards, as well as joint workflow coordination and preparation procedures with local staff. The helicopter was stationed within a maximum of 5 min flying distance from one of the comprehensive stroke centres. Specific materials, such as stent-retrievers, aspiration catheters, guiding catheters and others, were brought by the team for each intervention. Once the indication for EVT had been established, the pilot and the intervention team were alerted and prepared for takeoff. While the team was in transit, local hospital staff prepared the patient in the angiography room. Procedural steps were defined in site-specific protocols for each participating hospital. EVT was performed under general anaesthesia as per protocol. After EVT, patients were treated at the local intensive care unit or stroke unit until discharge.

During transfer weeks, once EVT was indicated, the emergency medical service was alerted, and the patient was transferred via helicopter or ambulance with the same priority as the prehospital stroke code alerts. Treatment procedures in the comprehensive stroke centres were organised at the discretion of the referral centres.

### Outcomes

The primary outcome of this analysis was the distribution of mRS scores 12 months after enrolment. The mRS is a scale of disability with possible scores ranging from 0 [no deficit] to 6 [death]. Scoring was carried out via a standardised, structured telephone interview after 12 months.^10^ Interviewers were certified and blinded to the patients’ allocation to the groups. If patients were not available via telephone, they received a questionnaire by mail and were requested to complete and return it.

Further secondary outcome parameters included dichotomised analyses of the mRS score, patient-rated EuroQol 5-dimensional questionnaire (EQ-5D-5L and the EQ-5D descriptive system describing mobility, self-care, usual activities, pain/discomfort and anxiety),^11^ Barthel index (scores ranging from 0 to 100, with the best score being 100),^12^ medical incidents and new diagnoses of diseases (eg, new ischaemic strokes, severe cardiovascular events, epilepsy, depression), rehospitalisation, in-hospital death and death within 12 months.

### Statistical analyses

Categorical variables are shown with absolute and relative frequencies, whereas ordinal data (mRS, Barthel index) and continuous data are shown as medians and IQRs. Ordinal dimensions of the EQ-5D-5L are shown as medians and IQRs and as percentages. Patients who were lost to follow-up were excluded from all analyses. No imputation methods were applied for missing data regarding baseline characteristics and outcomes. Categorical variables were compared via unadjusted and adjusted logistic regression. Ordinal logistic regression models (unadjusted and adjusted) were applied to analyse the mRS score, Barthel index score and single EQ-5D-5L dimensions. The adjusted common odds ratio (with corresponding 95% CIs) was used to present a shift in the direction of better outcome. For ordinal logistic regression of the mRS score, scores of 5 (severe disability) and 6 (death) were combined into a single “worst” category.^13^ The assumption of proportional odds was tested via the Brant test. Binary logistic regression models were used to calculate odds ratios for binary secondary outcomes. To estimate the mean difference in the EQ-5D-5L index between both groups, linear regression was applied. EQ-5D-5L index scores were obtained by using the German value set by Ludwig et al.^14^ Owing to heteroscedasticity and non-normally distributed residuals, the HC4 estimator for robust standard errors was used in the linear regression of the EQ-5D-5L index. In a sensitivity analysis, we used quantile regression to estimate the median difference in the EQ-5D index between groups.^15^ Both EQ-5D-5L index score and Barthel index score were analysed for surviving patients. In a sensitivity analysis, deceased patients were included in the EQ-5D-5L index by assigning a score of 0^16^ and in the Barthel index by assigning a score of −5. The adjustment parameters for the multivariable models were age, sex, NIHSS score and occlusion site (M1, M2, ICA, ICA with M1 involvement or basilar artery occlusion). Two-sided *P* values were used, and the level of significance was defined as < .05. Transfer served as the reference group in all regression analyses. For clarity, odds ratios and common odds ratios were oriented such that an odds ratio greater than 1 consistently indicates a more favourable outcome in the flying team group, whereas values less than 1 indicate a less favourable outcome. No formal adjustment for multiple comparisons was performed for secondary outcomes, as these analyses were considered exploratory. For all statistical analyses, “R” was used in version 4.4.1 (R Foundation for Statistical Computing).

## Results

**Figure 1:**
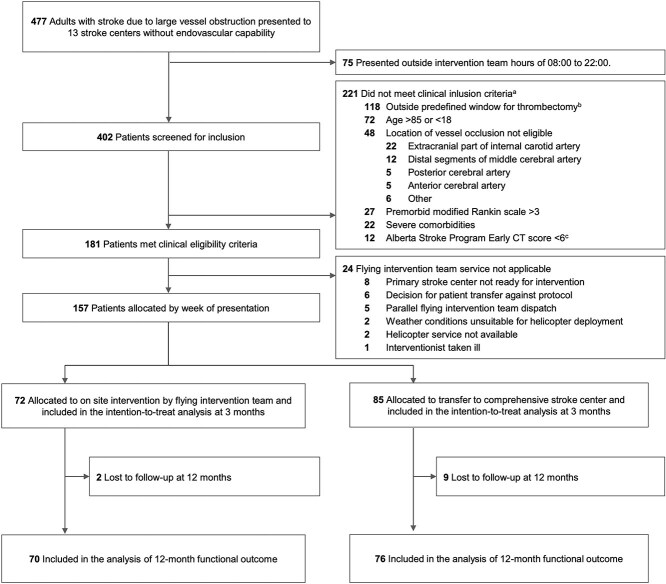
CONSORT flow diagram. (a) Multiple exclusion criteria are possible; patients may appear in more than one category. (b) Time window: > 6 h for anterior circulation occlusion without advanced imaging at the primary centre; > 24 h for posterior occlusion or anterior occlusion with mismatch on advanced imaging. (c) Alberta Stroke Program Early CT Score (ASPECTS): 10-point score of early ischaemic changes in MCA stroke; 1 point was deducted per affected region (0 = all affected, 10 = none affected). (d) Clinical improvement and/or spontaneous recanalisation.

Between 1 February 2018 and 24 October 2019, 157 patients were enrolled in the primary analysis. The final follow-up was 19 October 2020. Eleven patients were lost to follow-up; therefore, 146 patients were included in the secondary analysis at 12 months. Among these patients, 70 were treated by the flying team, and 76 were treated by patient interhospital transfer (Figure 1).

Baseline characteristics are shown in Table 1. Median age was similar between the groups (76 [IQR, 67–81] years in the flying team group vs 75 [66–79] years in the transfer group). Women were more frequent in the flying team group (64% vs 45%) and stroke severity was higher (NIHSS 15 [9–18] vs 13 [9–18]). Cardiovascular risk factors were similarly distributed, although prior myocardial infarction was more frequent in the transfer group (15% vs 6%). The distribution of occlusion sites was similar, with M2 occlusions occurring more frequently in the flying team group (29% vs 18%) and basilar artery occlusions occurring more frequently in the transfer group (9% vs 17%). The proportion of patients receiving intravenous thrombolysis treatment prior to EVT was similar (69% vs 70%).

**Table 1:** Demographics and baseline characteristics.

	**Flying team**	**Transfer**
**No. of patients**	70	76
**Demographics**		
** Age, median (IQR), y**	76 (67–81)	75 (66–79)
** Sex at birth**		
** Women, no. (%)**	45 (64)	34 (45)
** Men, no. (%)**	25 (36)	42 (55)
**Medical history, no. (%)**		
** Hypertension^a^**	46 (67)	53 (70)
** Atrial fibrillation**	22 (33)	23 (31)
** History of stroke**	18 (26)	15 (21)
** Diabetes mellitus^a^**	16 (23)	13 (17)
** History of myocardial infarction**	4 (6)	11 (15)
**Index stroke**		
** NIHSS, median (IQR)^b^**	15 (9–18)	13 (9–18)
** NIHSS groups, no. (%)^b^**		
** Mild stroke (NIHSS ≤ 5)**	7 (10)	8 (11)
** Moderate stroke (NIHSS 6–15)**	31 (44)	42 (55)
** Severe stroke (NIHSS > 15)**	32 (46)	26 (34)
** Intravenous thrombolysis treatment, no. (%)**	48 (69)	53 (70)
**Occlusion site, no. (%)**		
** MCA, M1 segment**	33 (47)	34 (45)
** MCA, M2 segment**	20 (29)	14 (18)
** Basilar artery**	6 (9)	13 (17)
** ICA**	6 (9)	8 (11)
** ICA with M1 involvement (“carotid T”)**	5 (7)	7 (9)
**Aetiology, no. (%)** ^**c**^		
** Macroangiopathy**	16 (23)	15 (20)
** Cardiac embolism**	41 (59)	38 (50)
** Unknown source**	10 (14)	21 (28)
** Other known source**	2 (3)	2 (3)

Abbreviations: ICA, internal carotid artery; no., number.

^a^Assessment of medical history by medical personnel during hospital stay.

^b^Evaluation of acute stroke (score range, 0–42; greater values indicate greater severity) with the NIHSS by telemedical examination or onsite neurologist.

^c^Aetiology was determined according to the TOAST (Trial of Org 10172 in Acute Stroke Treatment) classification, which categorises ischaemic stroke into large-artery atherosclerosis, cardioembolism, small-vessel occlusion, stroke of other determined aetiology and stroke of undetermined aetiology.

Workflow and procedural characteristics in the 12-month analysis cohort were consistent with those reported in the primary publication.^9^ Among patients who underwent EVT, median decision-to-puncture time was 58 min (IQR, 51–71) in the flying team group compared with 147 min (IQR, 122–185) in the transfer group (*P* < .001). Successful reperfusion (mTICI ≥ 2b) was achieved in 92% of patients in the flying team group and 82% in the transfer group (*P* = .139). Median puncture-to-recanalisation time was 36 min (IQR, 26–46) vs 44 min (IQR, 27–57), respectively (*P* = .141). Intracranial perforation occurred in 2% vs 10% (*P* = .058), distal embolisation in 7% vs 2% (*P* = .235) and arterial dissection in 2% vs 0% (*P* = .355).

### Primary outcome

**Figure 2:**
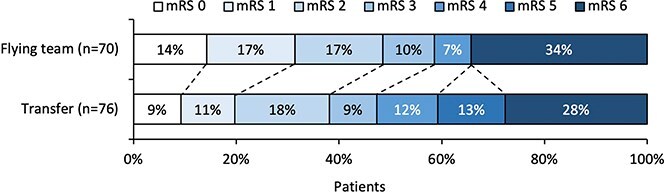
Distribution of the mRS score, with possible scores ranging from 0 (no deficit) to 6 (death), with scores of 5 and 6 being categorised together as a single category in the ordinal logistic regression model 12 months after EVT. The adjusted common odds ratio was 2.03 (95% CI, 1.09–3.84; *P* = .029), indicating a shift towards better functional outcome in the flying team group at 12 months. Multivariable models were adjusted for age, sex, NIHSS score and occlusion site. Abbreviation: EVT = endovascular thrombectomy.

At 12 months, the median mRS score was 3 [IQR 1–6] in the flying team group and 4 [2–6] in the control group. Ordinal logistic regression revealed a shift in the distribution of mRS scores in favour of the flying team group (adjusted common odds ratio [acOR], 2.03; 95% CI, 1.09–3.84; *P* = .029) (Figure 2; Table 2). The assumption of proportional odds was met (*P* adjusted = .23).

**Table 2:** Primary and secondary outcomes.

**Variable**	**Flyingteam (n = 70)**	**Transfer (n = 76)**	**Effect measure**	**Unadjustedvalue (95% CI)**	***P* value**	**Adjusted value(95% CI)**	***P* value**
**Primary outcome: mRS**							
** mRS, median (IQR)**	3 (1, 6)	4 (2, 6)	cOR	1.52 (0.84–2.75)	.160	2.03 (1.09–3.84)	.029
**Secondary outcomes**							
** Excellent outcome (mRS 0–1), no. (%)**	22 (31)	15 (20)	OR	1.86 (0.88–4.04)	.107	2.62 (1.11–6.50)	.031
** Good outcome (mRS 0–2), no. (%)**	34 (49)	29 (38)	OR	1.53 (0.79–2.97)	.205	2.08 (0.99–4.49)	.055
** Moderate outcome (mRS 0–3), no. (%)**	41 (59)	36 (47)	OR	1.57 (0.82–3.04)	.176	1.95 (0.95–4.08)	.071
** EQ-5D index, mean (SD)**	0.79 (0.24)	0.68 (0.31)	Beta	0.10 (−0.02–0.22)	.090	0.12 (0.00–0.24)	.0496
** EQ-5D-5L: mobility, median (IQR)**	2 (1, 3)	2 (1, 4)	cOR	1.52 (0.73–3.18)	.263	1.58 (0.73–3.45)	.249
** EQ-5D-5L: self-care, median (IQR)**	1 (1, 3)	2 (1, 4)	cOR	2.59 (1.15–5.80)	.022	3.03 (1.33–7.13)	.011
** EQ-5D-5L: usual activities, median (IQR)**	2 (1, 3)	3 (1, 4)	cOR	2.63 (1.24–5.61)	.013	3.08 (1.41–6.91)	.007
** EQ-5D-5L: pain/discomfort, median (IQR)**	1 (1, 2)	1 (1, 2)	cOR	1.33 (0.57–3.10)	.507	1.22 (0.48–3.08)	.670
** EQ-5D-5L: anxiety/depression, median (IQR)**	1 (1, 2)	1 (1, 2)	cOR	0.90 (0.40–2.03)	.806	1.04 (0.45–2.45)	.922
** Barthel index, median (IQR)**	95 (85–100)	95 (61–100)	cOR	1.39 (0.67, 2.88)	.379	1.99 (0.92–4.40)	.083
**Safety outcomes**							
** Death at 12 months, no. (%)**	24 (34)	21 (28)	OR	0.73 (0.36–1.48)	.385	0.79 (0.37–1.71)	.550
** Death during initial hospitalisation, no. (%)**	13 (19)	15 (20)	OR	1.08 (0.47–2.49)	.858	1.17 (0.49–2.85)	.727
** Bad outcome (mRS 5–6), no. (%)**	24 (34)	31 (41)	OR	1.32 (0.68–2.60)	.418	1.54 (0.74–3.27)	.253
**Medical incidents after initial hospitalisation**							
** No. of patients discharged**	57 (81)	61 (80)					
** Renewed stroke, no. (%)**	7 (13)	3 (5.5)	OR	0.37 (0.08–1.42)	.168	0.39 (0.08–1.62)	.215
** Severe cardiac conditions, no. (%)**	3 (5.8)	9 (16)	OR	3.20 (0.89–15.07)	.096	3.13 (0.82–15.33)	.115
** Epilepsy, no. (%)**	1 (1.9)	3 (5.5)	OR	2.94 (0.36–60.55)	.357	2.33 (0.20–58.12)	.520
** Depression, no. (%)**	3 (5.8)	7 (13)	OR	2.33 (0.61–11.31)	.239	2.29 (0.54–12.00)	.280
** Infection, no. (%)**	10 (19)	14 (25)	OR	1.37 (0.55–3.50)	.503	1.69 (0.64–4.68)	.296
** Accidents/falls, no. (%)**	4 (7.8)	7 (13)	OR	1.71 (0.48–6.90)	.414	2.41 (0.61–10.87)	.220
** Other diseases/events, no. (%)**	13 (25)	10 (18)	OR	0.65 (0.25–1.64)	.367	0.76 (0.28–2.01)	.581
** New hospital stay, no. (%)**	19 (37)	27 (49)	OR	1.62 (0.75–3.56)	.220	1.64 (0.73–3.75)	.235

Abbreviation: cOR, common odds ratio.

The outcomes were analysed via nonadjusted and adjusted ordinal logistic regression models. Multivariable models were adjusted for age, sex, NIHSS score and occlusion site. Scores on the mRS range from 0 to 6, with 0 indicating no symptoms, 1 indicating no clinically significant disability, 2 indicating slight disability, 3 indicating moderate disability, 4 indicating moderately severe disability, 5 indicating severe disability and 6 indicating death. In the regression analyses, severe disability (5) and death (6) were combined into a single worst category. The adjusted common odds ratio for a shift in the direction of a better outcome with the corresponding 95% CI is presented as an effect estimate. Unadjusted and adjusted odds ratios for good and bad outcomes, mortality and medical incidents were calculated via binary logistic regression models. Transfer served as the reference group in all analyses. For clarity, odds ratios and common odds ratios were oriented such that an odds ratio greater than 1 consistently indicates a more favourable outcome in the flying team group, whereas values less than 1 indicate a less favourable outcome.

### Secondary outcomes

Rates of excellent outcome (mRS 0–1) were 31% in the flying team group and 20% in the transfer group, with an adjusted odds ratio (aOR) of 2.62 (95% CI, 1.11–6.50; *P* = .031). Good outcome (mRS 0–2) was observed in 49% vs 38% (aOR, 2.08; 95% CI, 0.99–4.49; *P* = .055), and moderate outcome (mRS 0–3) in 59% vs 47% (aOR, 1.95; 95% CI, 0.95–4.08; *P* = .071).

**Figure 3:**
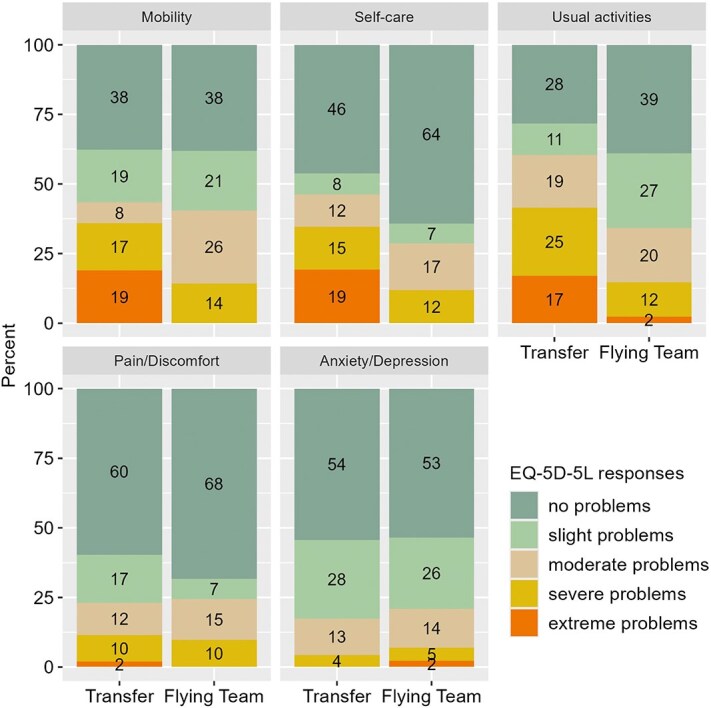
Distribution of responses in 5 EQ-5D-5L dimensions (mobility, self-care, usual activities, pain/discomfort and anxiety/depression) at 12 months after stroke, shown separately for patients treated by FIT and those transferred to a comprehensive stroke centre. Abbreviation: FIT = Flying Intervention Team.

For quality of life of surviving patients, adjusted linear regression of the EQ-5D-5L index revealed a better mean value in the flying team group (0.79 [SD, 0.24]) than in the transfer group (0.68 [0.31]), although the CI included values close to the null (β = 0.12; 95% CI, 0.00–0.24; *P* = .0496) (Table 2; Figure 3; Figure S2). As a robustness check, quantile regression confirmed this result (Table S1). When deceased patients with an assigned value of 0 on the EQ-5D index were included, the median scores were 0.58 [IQR, 0.00–0.89] in the flying team group and 0.48 [0.00–0.87] in the transfer group, with no significant adjusted difference (β = 0.04; 95% CI, −0.10 to 0.18; *P* = .584). Analyses of EQ-5D-5L dimensions favoured the flying team group for self-care (acOR, 3.03; 95% CI, 1.33–7.13; *P* = .011) and usual activities (acOR, 3.08; 95% CI, 1.41–6.91; *P* = .007). For the analysis of usual activities, the proportional odds assumption was not met, but the model was nevertheless retained as a pragmatic summarizing approach. Sensitivity analyses using multiple binary logistic regressions with different cut points indicated more favourable results in the flying team group regarding the proportion of severe problems. For mobility, the adjusted common odds ratio was 1.58 (95% CI, 0.73–3.45; *P* = .249). For pain/discomfort and anxiety/depression, no group differences were observed.^17,18^

For activities of daily living, the Barthel index of surviving patients had median values of 95 (IQR 85–100) in the flying team group and 95 (61–100) in the transfer group (acOR, 1.99; 95% CI, 0.92–4.40; *P* = .083). In a sensitivity analysis including deceased patients, who were assigned a value of −5 on the Barthel index, median scores were 85 [IQR, −5 to 100] vs 75 [−5 to 100], with no significant adjusted difference (acOR, 1.09; 95% CI, 0.59–2.04; *P* = .781) (Table 2; Figure S3; Table S1).

Regarding safety, no significant difference was seen in mortality between the flying team group (24 [34%]) and the transfer group (21 [28%]), with an aOR of 0.79 (0.37–1.71; *P* = .550). Survival analysis after 12 months revealed no significant differences (adjusted hazard ratio = 1.15 [0.63–2.09]; *P* = .659). Kaplan–Meier curves suggest that the higher mortality in the flying team group was mainly due to deaths occurring after 6 months (Figure S4). Death during initial hospitalisation occurred in 19% vs 20% of patients (aOR, 1.17; 95% CI, 0.49–2.85; *P* = .727). The proportion of patients with poor outcome (mRS 5–6) was 34% vs 41% (aOR, 1.54; 95% CI, 0.74–3.27; *P* = .253).

No significant differences were found in patient-reported medical incidents or new hospitalisations during follow-up, including recurrent stroke, cardiac conditions, epilepsy, depression, infections or accidents/falls (Table 2).

## Discussion

The use of the Flying Intervention Team to perform thrombectomy was associated with a more favourable distribution of long-term functional outcomes compared with patient interhospital transfer in nonurban areas.

This association may relate to the shorter time to treatment observed in the intervention group, a factor repeatedly shown to be strongly associated with functional outcome.^2^ The time advantage of the flying team can be explained by two main factors. First, the processes are carried out in parallel rather than consecutively. While the team is approaching, the patient is already being prepared in the angiography suite, allowing the procedure to start immediately upon arrival. In contrast, patient transfer requires a sequence of steps, including ambulance dispatch, patient preparation for transport, transport itself, reevaluation at the referral centre and preparation in the angiography suite. Second, most processes in the FIT model are concentrated within a single team, enabling rapid adjustments and short communication pathways. In contrast, patient transfer involves multiple actors, such as dispatch centres, emergency medical service and referral centres. In addition, the flying interventionists have a high individual case load through their interventional practice in both the FIT project and their own centre, which may improve the effectiveness of the intervention. Associations between individual physician volume (rather than hospital volume) and mortality or complications have been reported in other areas of medicine, including neurosurgical and endovascular procedures.^19–21^

Quality of life of survivors, as assessed with the EQ-5D score, was better in the flying team group, mainly because of improvements in self-care, activities of daily living and mobility. In contrast, no effect was observed on pain/discomfort or anxiety/depression, domains that may be less time dependent than motor function in the treatment of stroke. Interpretation of these findings is limited by the high mortality observed in both groups. To address this, we conducted a sensitivity analysis in which deceased patients were assigned a value of 0, thus combining survival and quality of life into one measure as proposed in previous studies. With this approach, no significant differences between groups were observed.

Among surviving patients, results for activities of daily living were directionally similar to the primary outcome but did not meet statistical significance. A well-known limitation of the Barthel index is its ceiling effect. In our population, more than one-quarter of patients reached the maximum score of 100, despite some having residual symptoms. As a result, differences between groups within these upper 25% could not be detected with the Barthel index.

Overall mortality was high at 12 months. A greater proportion of patients died in the flying team group (34% vs 28%). However, the proportion of patients with severe disability (mRS 5) was higher in the transfer group (0% vs 13%). Overall, “poor outcome” of mRS scores of 5 and 6 was lower in the flying team group, despite a 2-point higher median NIHSS score at baseline. None of the predefined post-discharge incidents were significantly different between the two groups, suggesting that there were no critical differences in safety after hospital stay.

Beyond the clinical findings, the organisational and economic implications of this model merit consideration. Implementation of the FIT model requires substantial logistical coordination and financial resources, including helicopter transport, availability of specialised neurointerventionalists and preparedness of local angiography suites. These organisational demands may limit feasibility in regions without established telemedicine networks and pre-existing infrastructure.

In addition to direct transport and personnel costs, opportunity costs related to the temporary absence of interventionalists from their primary centres should be considered. At the same time, the model may reduce secondary transfer-related costs, prolonged transport times and potential downstream costs associated with treatment delay. A formal cost-effectiveness analysis was beyond the scope of the present study. Future research should evaluate economic sustainability and scalability of this approach compared with conventional interhospital transfer models.

Our study has several limitations. First, this is a secondary analysis of a controlled intervention study, the limitations of which have been described previously.^9^ It therefore allows the generation of hypotheses but does not prove causal effects. Second, the study was not powered to detect differences in long-term functional outcome, as time delay was the primary outcome of the original study. Third, outcome was assessed via telephone, and although the certified interviewers were blinded to the treatment group, unblinding may have occurred during the interviews. To minimise this risk, the mRS was scored before other questions were answered. Furthermore, bias could occur if patients were unable to respond themselves and if next-of-kin were interviewed. Fourth, functional outcome may have been influenced by unknown or unmeasurable confounders. Fifth, basilar artery occlusions were more frequent in the transfer group. Although occlusion site was adjusted for in all multivariable models, the distinct clinical course of posterior circulation stroke may limit complete statistical control, and residual confounding cannot be excluded. Sixth, multiple secondary and exploratory outcomes were analysed without adjustment for multiplicity. These findings should therefore be interpreted as exploratory. Seventh, interpretation of the patient-reported outcomes EQ-5D and Barthel index is limited by high mortality rates in both groups. By presenting both survivor-only results and sensitivity analyses including deceased patients with assigned fixed values, we aimed to maximise the interpretability and transparency of patient-reported outcomes. Eighth, generalisability may be limited, as the study was conducted in nonurban regions in Germany, with telemedicine-supported stroke care, and findings may not directly apply to other healthcare systems. In addition, 11 patients were lost to follow-up at 12 months. Although this represents a small proportion of the cohort, exclusion of patients with missing follow-up data may introduce bias. Furthermore, predefined eligibility criteria, including the upper age limit of 85 years and restricted intervention hours, were inherent to the original study design and may limit generalisability beyond the studied population. Finally, confidence intervals around some of the effect estimates were wide, reflecting limited sample size and high variability and should be interpreted with caution.

## Conclusion

In this study, the Flying Intervention Team was associated with improved functional outcomes at 12 months in patients with LVO. These findings are consistent with the previously reported 3-month outcomes and suggest that the observed differences were maintained over time. Further research should evaluate generalisability and integration into various healthcare systems.

## Supplementary Material

aakag025_Supplementary_Material_One-Year_Functional_Outcome_FIT

## Data Availability

Data used for this study are available from the authors upon reasonable request.
