# Supplementary material for: One-year functional outcome of the Flying Intervention Team versus patient interhospital transfer in acute ischaemic stroke
Source: Eur Stroke J. 2026 Apr 6;11(4):aakag025. doi: 10.1093/esj/aakag025 (PMC13058261; doi:10.1093/esj/aakag025)
Supplement: aakag025_Supplementary_Material_One-Year_Functional_Outcome_FIT [file aakag025_supplementary_material_one-year_functional_outcome_fit.pdf]

Supplementary Material

One-Year Functional Outcome of the Flying Intervention Team versus Patient Interhospital Transfer in Acute Ischemic Stroke

Nikolai D Hubert, Markus Holler, Saskia R Wernsdorf, Sophie Herdegen, Christian Maegerlein, Hanni Wiestler, Lucie Esterl-Pfäffl, Dennis Dietrich, Thomas Witton-Davies, Isabel Heinrich, Anastasios Mpotsaris, Philip M Bath, Heinrich J Audebert, Roman L Haberl, Gordian J Hubert.

Table of contents

Figure S1: Map of the TEMPiS network and its operational area in Southeast Bavaria

Figure S2: Boxplot of the EQ-5D-5L index scores at 12 months

Figure S3: Boxplots of the Barthel index at 12 months

Figure S4: Survival at 12 months

Table S1: Sensitivity analyses of secondary outcome variables

Figure S1: Map of the TEMPiS network and its operational area in Southeast Bavaria

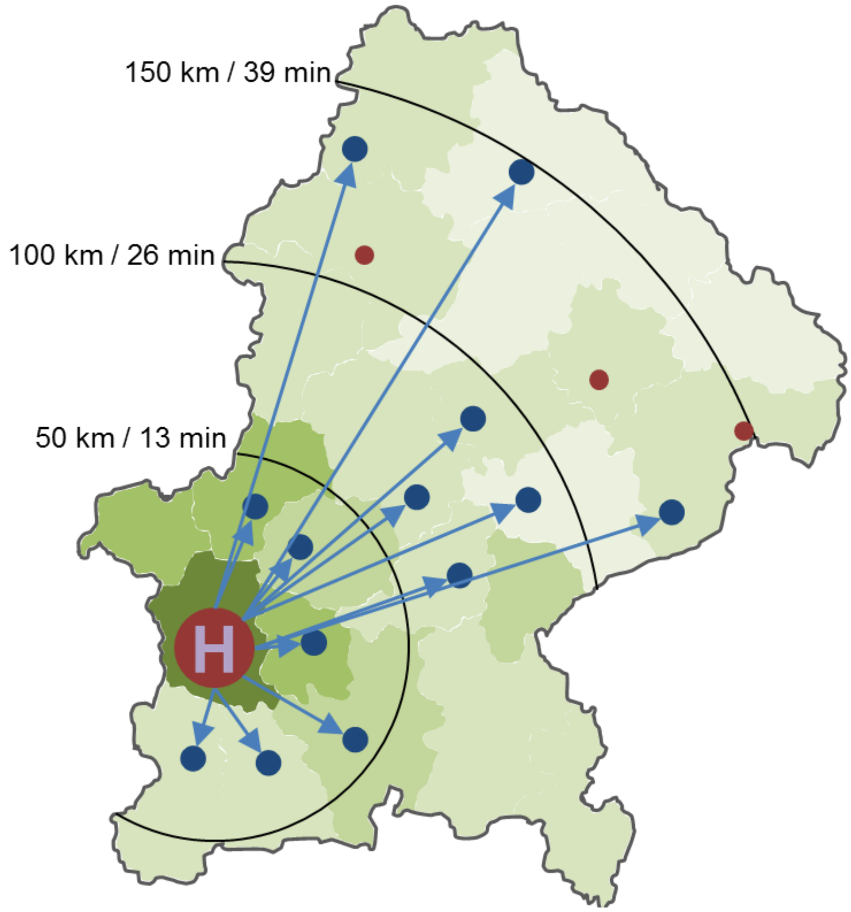

Primary stroke centres taking part in the FIT project are shown as blue dots. The referral centres for interhospital transfers are visible as small red dots. The helicopter site is shown as a large red dot with “H”, which represents two nearby referral centres in Munich. Flight routes are shown as blue arrows.
A, All primary stroke centres receive Flying Intervention Team service from Munich. The flight distance and flying time in minutes are given at each black half-circle.
B, Transfer directions and transfer time estimates from each primary stroke centre to the preferred referral centres are shown. Transfer time estimates were calculated considering general proportions of airborne and ground transfer, a helicopter flying speed of 220 km/h, and estimates of driving times on optimal routes according to the route planner service of the General German Automobile Club (http://maps.adac.de).
Reproduced with permission from Hubert et al., JAMA. 2022; doi:10.1001/jama.2022.5948. © American Medical Association. All rights reserved, including those for text and data mining, AI training, and similar technologies.

Figure S2: Boxplot of the EQ-5D-5L index scores at 12 months

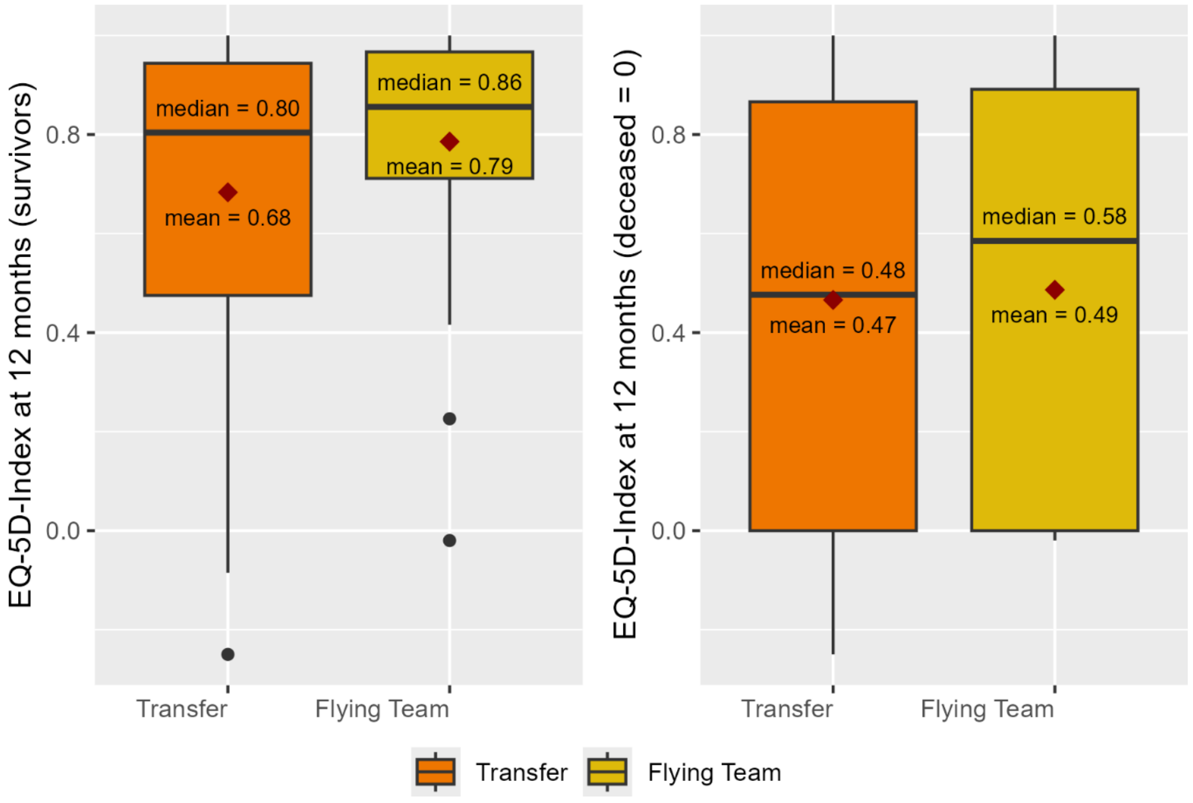

Boxplots show median and mean values of the EQ-5D-5L index for survivors (left panel) and for all patients, including deceased individuals with an index score of 0 (right panel).

Figure S3: Boxplots of the Barthel index at 12 months

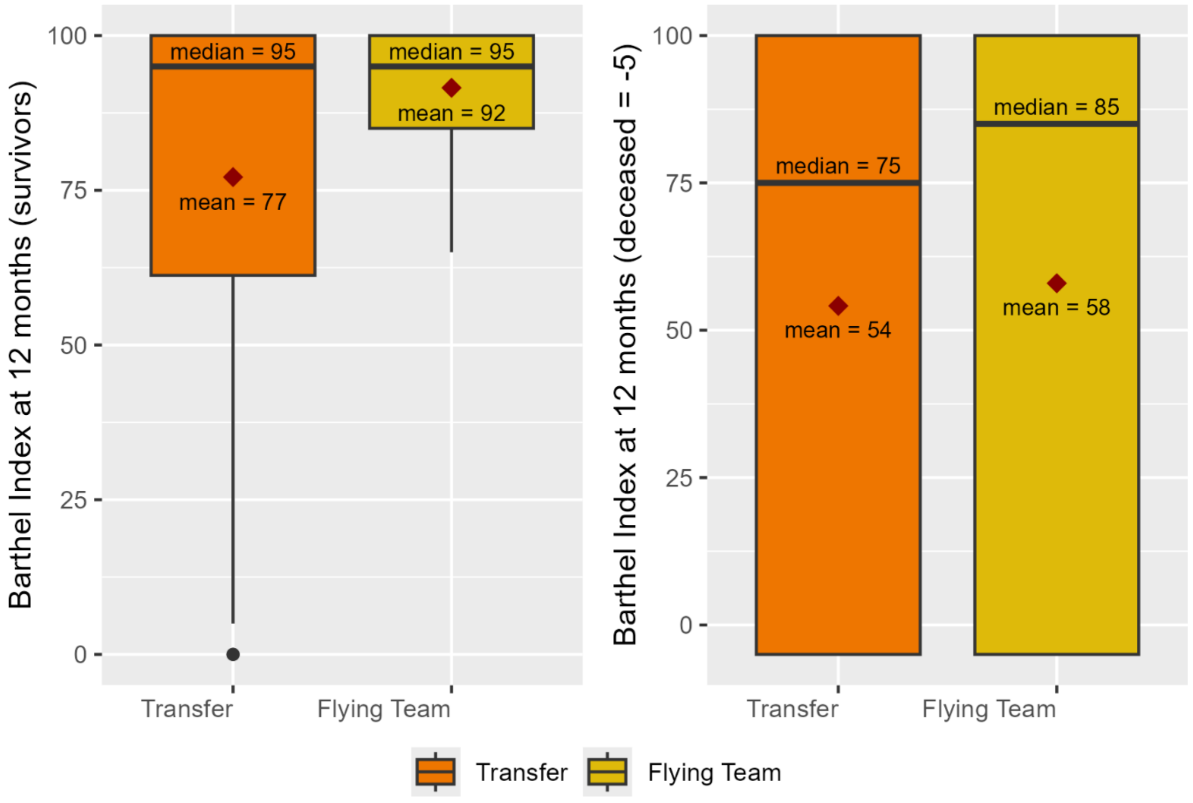

Boxplots show median and mean values of the Barthel index for survivors (left panel) and for all patients, including deceased individuals with an index score of -5 (right panel).

Figure S4: Survival at 12 months

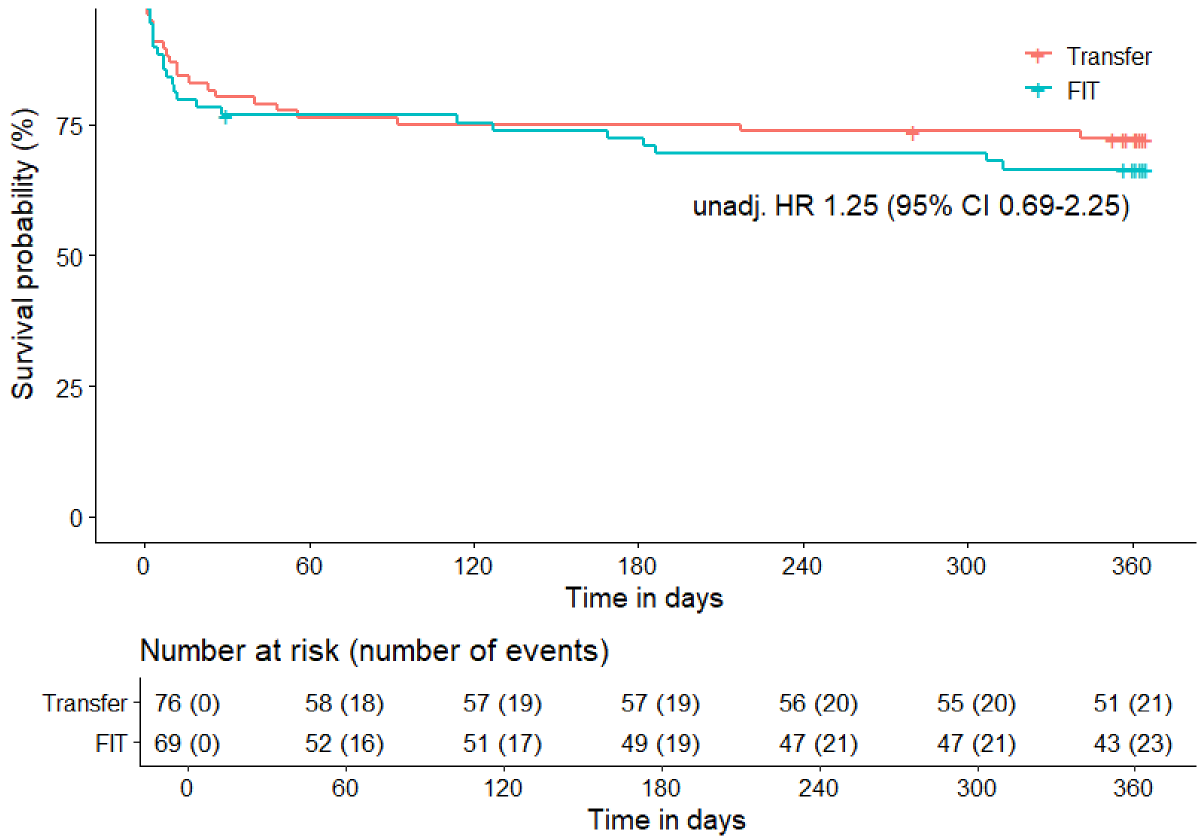

Kaplan-Meier survival curves for the flying team group and the transfer group. Abbreviations: FIT = Flying Intervention Team; CI = confidence interval.

Table S1: Sensitivity analyses of secondary outcome variables

Variable	Flying Team (n = 70)	Transfer (n = 76)	Effect Measure	Unadjusted Value (95%-CI)	P value	Adjusted Value (95%-CI)	P value
**Quantile Regression of EQ-5D index**							
EQ-5D index (survivors), median (IQR)	0.86 (0.71, 0.97)	0.80 (0.47, 0.94)	Cond. median	0.05 (-0.034, 0.22)	NA[a]	0.07 (0.05, 0.247)	NA
EQ-5D index (incl. deceased patients), median (IQR)	0.58 (0.00, 0.89)	0.48 (0.00, 0.87)	Cond. median	0.11 (-0.124, 0.356)	NA[a]	0.12 (-0.222, 0.313)	NA
**Logistic regressions of EQ-5D-5L: Usual activities with different cut points**							
Usual activities 1 (no problems) vs. 2-5, No. (%)	16 (39%)	15 (28%)	OR	1.62 (0.68, 3.89)	0.274	1.86 (0.69, 5.16)	0.222
Usual activities 1-2 vs. 3-5, No. (%)	27 (66%)	21 (40%)	OR	2.94 (1.28, 7.01)	0.013	3.29 (1.37, 8.33)	0.009
Usual activities 1-3 vs. 4-5, No. (%)	35 (85%)	31 (58%)	OR	4.14 (1.56, 12.45)	0.007	4.67 (1.68, 14.87)	0.005
Usual activities 1-4 vs. 5 (extreme problems), No. (%)	40 (98%)	44 (83%)	OR	8.18 (1.44, 154.26)	0.051	12.41 (1.60, 375.41)	0.051
**Additional regression models including deceased patients**							
EQ-5D index (incl. deceased), mean (SD)	0.49 (0.43)	0.47 (0.41)	Beta	0.02 (-0.13, 0.17)	0.781	0.04 (-0.10, 0.18)	0.584
Barthel Index (incl. deceased), median (IQR)	85 (-5, 100)	75 (-5, 100)	cOR	0.93 (0.52, 1.67)	0.811	1.09 (0.59, 2.04)	0.781
**Survival**							
Death at 12 months, No. (%)	24 (34%)	21 (28%)	HR	1.25 (0.69, 2.25)	0.467	1.15 (0.63, 2.09)	0.659

Abbreviations: CI, confidence interval; Cond. median, Conditional median; IQR, interquartile range. Transfer served as the reference group in all analyses.
[a] The rank inversion method used for computing the confidence intervals does not provide a p-value
